# Anti-Inflammatory and Synaptic Protective Effects of TNF-α Inactivation in the MDX Mouse Model

**DOI:** 10.3390/cimb48030270

**Published:** 2026-03-03

**Authors:** Anna Oller Bonani, Valquíria Matheus, Ana Laura Midori Rossi Tomiyama, Alexandre Leite Rodrigues de Oliveira

**Affiliations:** 1 Laboratory of Nerve Regeneration, Department of Structural and Functional Biology, Institute of Biology, UNICAMP, Campinas 13083-862, SP, Brazil; anna.bonani2@gmail.com (A.O.B.); analauratomiyama@gmail.com (A.L.M.R.T.); 2 Advanced Therapies Laboratory, Faculty of Pharmaceutical Sciences, UNICAMP, Campinas 13083-862, SP, Brazil; valmath@unicamp.br

**Keywords:** central nervous system, spinal motoneurons, Duchenne muscular dystrophy, MDX, synapse, tumor necrosis factor alpha

## Abstract

Background: Duchenne muscular dystrophy (DMD) is a severe neuromuscular disorder caused by the absence of functional dystrophin, leading to progressive muscle degeneration, inflammation, and alterations in the central nervous system. The sustained inflammatory response in DMD increases glial activation and the release of tumor necrosis factor alpha (TNF-α), which contributes to muscle fiber damage. Here, we investigated the anti-inflammatory and neuroprotective effects of Etanercept, a TNF-α receptor-blocking therapeutic, on the spinal cord of MDX mice, a widely used model of DMD. Methods: Adult male MDX and control C57BL/10 mice received vehicle or Etanercept (3, 6, or 12 mg/Kg, intraperitoneally (i.p.)) every 72 h for two weeks, along with daily gait assessment. At the end of treatment, flow cytometry and immunolabeling analyses were performed in the lumbar spinal cord. Results: Etanercept at 12 mg/Kg reduced astrogliosis and microglial activation; restored synaptic markers, including synaptophysin, glutamic acid decarboxylase 65 (GAD-65), and vesicular glutamate transporter 1 (VGLUT-1); and decreased pro-inflammatory cytokines. The treatment reduced GFAP+/TNF-α+ astrocytes and significantly downregulated Th1 lymphocyte polarization in treated MDX mice. These cellular effects were accompanied by improvements in locomotor function. Conclusions: Together, our findings indicate that TNF-α blockade by Etanercept exerts neuroprotective and anti-inflammatory actions in the spinal cord of dystrophic mice, providing new insights into the impact of TNF-α signaling on neuroinflammatory processes in DMD.

## 1. Introduction

Muscular dystrophies constitute a very extensive and heterogeneous group of genetic neuromuscular diseases characterized by progressive degeneration with muscle dysfunction and loss [1]. Duchenne muscular dystrophy (DMD) is one of the most severe and common forms, with an incidence of 1 in every 6500 individuals [2]. This disease has a recessive inheritance pattern, with a high rate of mutations in the *DMD* gene located on the short arm of the X chromosome, in the Xp21 region. Because it is an X-linked disorder, it rarely affects women, who are usually carriers of the mutated gene [3]. A large number of distinct mutations in the *DMD* gene have been identified in patients with Duchenne muscular dystrophy. These genetic alterations prevent the production of the functionally active muscle isoform of dystrophin (Dp427m). In severe cases, dystrophin expression is completely absent, whereas in others, truncated or structurally abnormal dystrophin proteins may be produced. In both situations, the lack of functional dystrophin compromises its normal role in anchoring cytoskeletal proteins to integral membrane proteins [4,5], resulting in sarcolemmal fragility, increased calcium influx, chronic inflammation, and progressive muscle fiber degeneration [6,7]. Although satellite cells initially promote regeneration, repeated cycles of degeneration ultimately impair repair and favor replacement of muscle by fat and fibrosis [8]. Clinically, DMD manifests in early childhood, with progressive muscle weakness, loss of ambulation, and later respiratory and cardiac failure [2,9,10]. The C57BL/10-mdx mouse is widely used as an experimental model of DMD, exhibiting elevated creatine kinase levels, inflammatory infiltration, fibrosis, and myopathic lesions, particularly between 4 and 6 weeks of age [6,11,12].

It is now understood that DMD goes beyond muscle damage, affecting other functions [8], including the nervous system. However, there are still few studies on this subject [13,14]. Cycles of denervation and reinnervation occur at the neuromuscular junctions of spinal alpha motoneurons innervating dystrophic fibers, leading to synaptic fragmentation and reorganization of pre- and postsynaptic elements [9,10]. In the spinal cord, motoneurons located in the ventral column establish motor output to skeletal muscle, and their function depends on tightly regulated interactions with surrounding glial cells [7,15]. Astrocytes and microglia play central roles in maintaining homeostasis and responding to pathological insults. Under inflammatory conditions, astrocytes become reactive, increasing GFAP expression, while activated microglia upregulate IBA-1, both contributing to neuroinflammatory processes that may impact neuronal function [16,17,18,19].

Inflammation is a central component of DMD pathology. Dystrophic muscles exhibit significant immune cell infiltration, with tumor necrosis factor alpha (TNF-α) playing a pivotal role in promoting myofibrillar breakdown [20,21]. In addition to its peripheral effects, TNF-α acts as a key mediator in immune-to-brain communication, modulating glial activation and neuronal excitability. Therefore, systemic inflammation associated with DMD may also influence central nervous system responses, suggesting that therapeutic strategies targeting TNF-α could have both muscular and neural implications [22].

Etanercept is an anti-inflammatory drug that acts as a competitive inhibitor of tumor necrosis factor alpha (TNF-α) and has been approved for the treatment of rheumatoid arthritis (RA) [23]. It is a recombinant human dimeric fusion protein that binds TNF-α through its extracellular receptor domain linked to the Fc portion of human IgG1, thereby preventing its interaction with cell surface receptors and inhibiting inflammatory signaling. In dystrophic models, Etanercept has been shown to reduce the degenerative effects of DMD muscle by blocking TNF-α, providing a prolonged protective effect [24]. The drug attenuated muscle necrosis; inflammatory infiltration—particularly neutrophils; and exercise-induced damage, in addition to lowering creatine kinase (CK) levels and preventing reductions in chloride channel gCl and potassium conductance [20,21].

**Figure 1:**
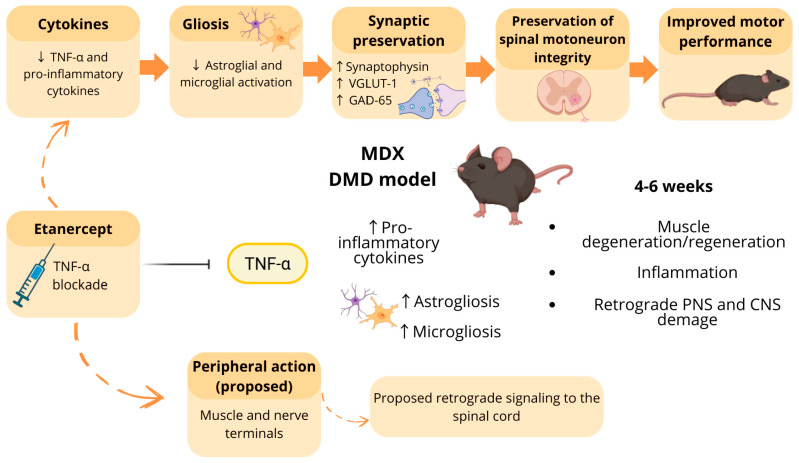
Proposed effects of TNF-α blockade by Etanercept on the muscle–nerve–spinal cord axis in MDX. In the MDX model, particularly between 4 and 6 weeks of age, increased levels of pro-inflammatory cytokines are associated with muscle inflammation, degeneration/regeneration cycles, and retrograde damage affecting both the peripheral nervous system (PNS) and the central nervous system (CNS), including astrogliosis and microgliosis in the spinal cord. Etanercept treatment promotes TNF-α blockade, leading to reduced pro-inflammatory cytokines and decreased glial activation. These experimentally observed effects are associated with synaptic preservation, evidenced by increased synaptophysin, VGLUT-1, and GAD-65 expression, as well as maintenance of spinal motoneuron integrity, potentially contributing to improved motor performance. Solid arrows indicate effects supported by experimental data, whereas dashed arrows represent proposed mechanisms, including the hypothesis that peripheral actions of Etanercept in muscle and nerve terminals trigger retrograde signaling toward the spinal cord, modulating central outcomes. Created in BioRender. Laura Midori Rossi Tomitama (2026). BioRender.com/p10iajd (accessed on 11 February 2026).

Despite consistent evidence of muscular benefit, the effects of Etanercept on the central nervous system (CNS) in DMD remain poorly explored. It is still unclear whether peripheral TNF-α inhibition can modulate central inflammatory responses and preserve synaptic components and functional aspects of the neuron–muscle axis involved in motor control. Considering the role of TNF-α in immune–brain communication and central immune activation [20], we hypothesized that TNF-α blockade with Etanercept would attenuate systemic and spinal cord inflammation, preserve synaptic integrity, and improve motor function (Figure 1).

In this context, the present study aimed to investigate, for the first time, the retrograde effects of Etanercept on the spinal cord, evaluating its action on motoneurons and surrounding glia, as well as its impact on synaptic preservation and reduction of Ca^2+^ and glutamate-mediated excitotoxicity.

## 2. Materials and Methods

### 2.1. Animals

C57BL/10 and MDX mice (males, 4 weeks old, 15–20 g) were obtained from the Multidisciplinary Center for Biological Research at the University of Campinas (CEMIB/UNICAMP, Campinas, SP, Brazil) and housed in the Laboratory of Nerve Regeneration’s animal facility at the Institute of Biology, UNICAMP. The animals were kept in mini-isolators (Alesco), with free access to food and water ad libitum, with a 12:12 h light–dark cycle, and kept in an acclimatized environment at 23 °C. The experiments were approved by the Ethics Committee for the Use of Animals (Institute of Biology-CEUA/IB/UNICAMP, protocol no. 5862-1/2021) and carried out in accordance with the guidelines of the Brazilian Committee for Ethics in the Use of Animals (CONCEA). The animals were organized into groups for an optimized evaluation by different techniques, groups, and experimental analyses, as detailed in Table 1.

### 2.2. Drug Treatment

Mice were treated with Etanercept at doses of 3, 6, or 12 mg/Kg. The vehicle group received 0.08% methyl cellulose solution alone. Etanercept was diluted in sterile phosphate-buffered saline (PBS) to achieve the appropriate dose for each animal in a fixed final injection volume of 100 µL. Treatments were administered intraperitoneally on experimental days 1, 3, 6, 9, and 12, totaling five doses per animal. All treatments began at the end of the fourth week of age and were administered in the morning, following behavioral testing. Vehicle-treated mice received injections at the same frequency and volume as the Etanercept-treated groups. Animals were euthanized on experimental day 14, corresponding to the end of the sixth week of life. Animals were grouped according to strain (MDX or C57BL/10) and treatment (vehicle or Etanercept at 3, 6, or 12 mg/Kg). Five animals per group were used for immunohistochemical analyses. Because both immunohistochemistry and flow cytometry require spinal cord tissue, separate cohorts of animals were used for each technique. Accordingly, an additional five animals per group were included for flow cytometry analysis, which was performed only in the vehicle and 12 mg/Kg Etanercept groups, as 12 mg/Kg was identified as the most effective dose.

### 2.3. Collection and Preparation of Tissue Samples

Mice were anesthetized with an overdose of xylazine (30 mg/Kg) and ketamine (300 mg/Kg) and underwent thoracotomy, followed by transcardial perfusion with phosphate-buffered saline (0.9% NaCl in phosphate buffer (PB), pH 7.4). Animals designated for immunohistochemistry and cytochemistry analyses were subsequently perfused with a fixative solution consisting of 4% paraformaldehyde in 0.1 M PB. Following fixation, the tibialis cranialis and soleus muscles, as well as the spinal cord, were dissected and immersed in the same fixative solution overnight at 4 °C. Samples were then washed three times in 0.1 M PB and immersed in a graded series of sucrose solutions for cryoprotection: 10%, 20%, and 30% sucrose for the spinal cord and muscles, and 40% and 50% sucrose for the muscles, each for 24 h. Subsequently, samples were embedded in Tissue-Tek, frozen in n-hexane at controlled temperatures (−25 to −30 °C for the spinal cord and −75 to −80 °C for the muscles), and stored at −20 °C (spinal cord) and −80 °C (muscles) until further processing.

### 2.4. Immunohistochemistry

Transverse sections, 12 μm thick, of the lumbar intumescence were obtained using a cryostat (Microm International GmbH, HM525, Walldorf, Germany), and they were then transferred to gelatinized glass slides and stored at −20 °C until use. For immunohistochemistry, slides were acclimatized at room temperature, and the sections were outlined with a hydrophobic pen (PAP pen, Sigma Z377821, Redding, CA, USA). The slides were then transferred to a humid chamber protected from light. The sections were immersed in 0.01 M PB (3 × 5 min each) and incubated with 100 μL of blocking solution (3% bovine serum albumin in 0.1 M PB) for 45 min. The primary antibodies (Table A1) were then diluted in an incubation solution (1.5% bovine serum albumin and 0.2% Tween in 0.1 M PB), and the sections were incubated for 4 h at room temperature. After incubation with the primary antibodies, the sections were washed with 0.01 M PB and incubated with the appropriate secondary antibody (Alexa Fluor 488) for 45 min. The sections were again washed with 0.01 M PB and mounted on a coverslip with glycerin/PB (3:1). The slides were observed under an epifluorescence microscope (Leica DMB5500, Wetzlar, Germany) and documented with a digital camera (Leica DFC345FX, Wetzlar, Germany), using specific filters according to the secondary antibodies. For quantification, three representative images were selected from each specimen in each experimental group. Quantification was then carried out using the integrated density of pixels, using ImageJ software (version 1.33u, National Institutes of Health, Bethesda, MD, USA). The arithmetic mean of the integrated density of pixels from each section (three sections per spinal cord) was calculated and averaged per group.

### 2.5. Flow Cytometry

After perfusion with PBS, the spinal cord L4, L5, and L6 lumbar segments were dissected. The tissue was dissociated, and astrocytes and lymphocytes were isolated using a Percoll density gradient, according to a previously established protocol [25]. Following cell isolation, cells were stimulated with phorbol 12-myristate 13-acetate (PMA), Ionomycin, and Brefeldin A for 4 h at 37 °C in a 5% CO_2_ incubator to prevent cytokine secretion and allow for intracellular immunolabeling.

After washing, cells were incubated with the antibodies listed in Table A2. Cells were then washed and fixed using commercial fixation and permeabilization buffers (eBioscience, San Diego, CA, USA), according to the manufacturer’s instructions, and subsequently analyzed on a NovoCyte flow cytometer (ACEA Biosciences, San Diego, CA, USA). Data analysis was performed using NovoExpress software (version 1.3.0; ACEA Biosciences, San Diego, CA, USA).

### 2.6. Muscle Cytochemistry

Hematoxylin and eosin (H&E) staining was used for the analysis of muscle morphology in longitudinally sectioned muscle samples prepared using a cryostat. Initially, the hematoxylin/eosin (H&E) stain was prepared. For this, 2 g of hematoxylin was dissolved in 100 mL of 95% ethanol, while 3 g of potassium alum was dissolved in heated water under continuous stirring. Subsequently, the hematoxylin solution was gradually added to the potassium alum solution under agitation. Then, 100 mL of glycerin and 10 mL of acetic acid were added inside a fume hood while stirring. The solution was filtered and stored in an amber bottle. For the counterstain, 1 g of Eosin Y was diluted in 200 mL of 95% ethanol and 1 mL of acetic acid. The solution was then filtered and stored in a dark bottle.

After the stain preparation, the slides were post-fixed in 4% paraformaldehyde (PFA) for 1 min, followed by immersion in the first stain (hematoxylin) for 3 min. The slides were then rinsed under running water for 3 min and immersed in the second stain (eosin) for 4 min. Finally, they were rinsed in distilled water, and the slides were sealed and mounted by covering the tissue with a glass coverslip using a mounting medium for fixation.

### 2.7. Assessment of Limb Strength

The evaluation of muscular function, specifically regarding limb strength, was performed using the Grip Strength Test. This test was conducted on MDX and control mice groups treated with Etanercept at different doses, as well as with the vehicle, for subsequent comparison.

Each animal was gently suspended by the tail, manually, so that the hind limbs were unsupported, inducing them to use their forelimbs to grasp a metal grid (1–2 mm in diameter) attached to a force transducer. The force exerted during traction was measured in three repetitions per day during the 13-day treatment period. The recorded values were expressed in grams. The average of the three measurements was calculated, and the data displayed on the screen were collected manually and entered into an Excel spreadsheet. Subsequently, statistical analyses were performed using ANOVA and the *t*-test to determine whether there were significant differences between the different treatment groups (GraphPad Prisma software—version 7.05).

### 2.8. Motor Function Test

Functional analysis was evaluated using an automated system (CatWalk System, Noldus Inc, Wageningen, The Netherlands), in which the animal walks spontaneously on a glass platform illuminated with a green light, and a red illumination on the top of the walkway enhances the contrast of the footprints. Three runs were recorded by a high-speed camera placed under the walkway, and the data were analyzed with CatWalk XT 10.6 software (Noldus Inc.). The sciatic function index (SFI) was calculated according to the following formula [26]: SFI = 118.9 (ETS − NTS)/NTS) − 51.2 (EPL − NPL)/NPL) − 7.5, where N—normal side; E—experimental side; PL—print length; and TS—toe extension. Additionally, the step sequence (calculation of total duration of all runs and the total number of categorized footfall patterns) and Max Contact Max Intensity (Maximum Intensity at Max Contact of a paw, which depends on the degree of contact between a paw and the glass plate) parameters were evaluated. Every day, from the beginning of the experiment, spontaneous walking footprints were collected for thirteen consecutive days, in the same period and under the same environmental conditions. The mice were allowed to move freely in both directions, with a running speed between 0.50 and 5 mm/s at a maximum permitted speed with a variation of 60%. The camera gain was set to 25.01, and the detection limit to 0.25. Three conforming runs were kept and analyzed.

### 2.9. Statistical Analysis

The mean and standard error of the mean (SEM) were calculated for each experimental group. One-way ANOVA was used to evaluate the immunohistochemistry results, followed by the Tukey post hoc test. Functional analysis (walking track test and grip strength) was assessed by two-way ANOVA, followed by the Tukey post hoc test. Paw strength analysis was performed using one-way ANOVA, followed by Tukey’s post hoc test, as well as *t*-test (parametric test) for daily analysis. Spinal cord flow cytometry was assessed using one-way ANOVA, followed by Tukey’s post hoc test. The following significance levels were adopted: *p* < 0.05 (*), *p* < 0.01 (**), *p* < 0.001 (***), and *p* < 0.0001 (****). GraphPad Prism software (v. 7.05) was used to carry out the analyses.

## 3. Results

The results are presented according to the effects of TNF-α blockade on inflammatory profiles, followed by glial reactivity in the spinal cord, preservation of synaptic inputs to motoneurons, skeletal muscle morphology, and culminating in functional motor outcomes. Quantitative immunohistochemical, flow cytometric, morphological, and functional analyses were used to assess dose-dependent responses to Etanercept treatment in comparison with vehicle-treated MDX and C57BL/10 control animals.

### 3.1. Modulation of Peripheral Inflammatory Markers by Etanercept Treatment

**Figure 2:**
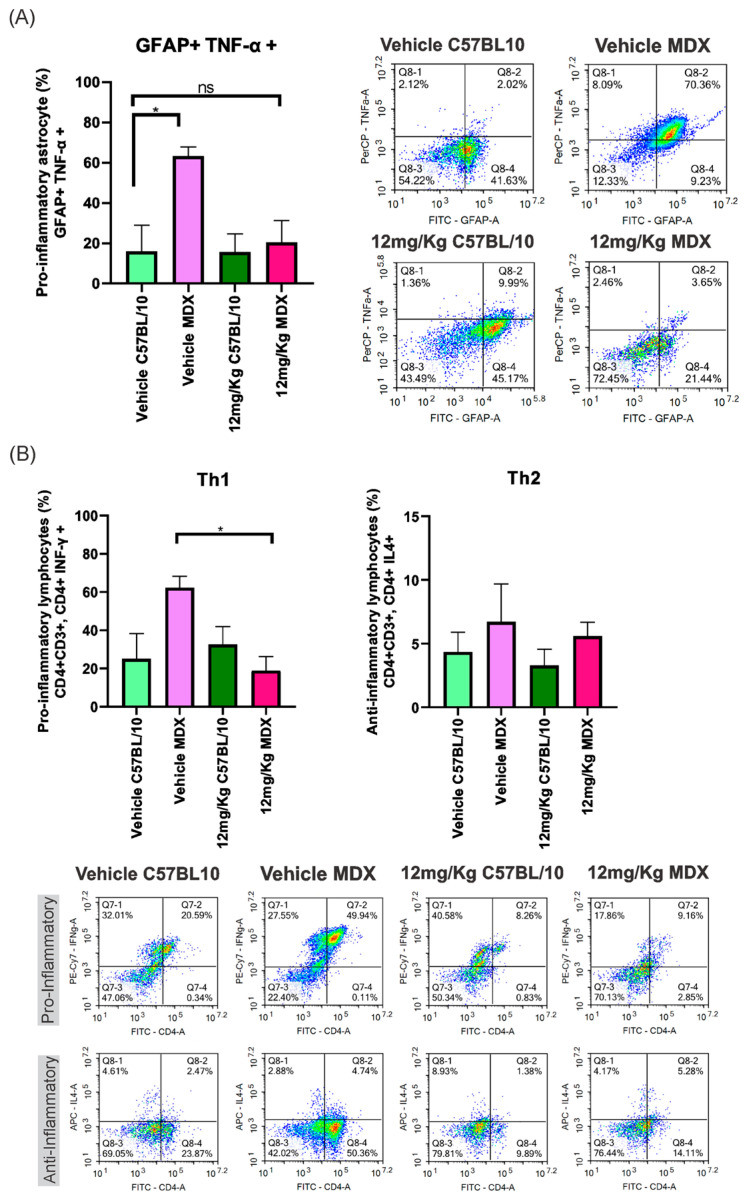
(**A**) The percentage of pro-inflammatory astrocytes (GFAP+; TNF-α+) in both strains (MDX and C57BL/10), comparing vehicle and Etanercept treatment at 12 mg/Kg. Dot plot graphs display the TNF-α levels in pro-inflammatory astrocytes in the vehicle groups and in those treated with Etanercept at 12 mg/Kg for both strains. (**B**) The percentage of pro-inflammatory and anti-inflammatory lymphocytes in both strains, comparing vehicle and treatment at 12 mg/Kg. Dot plot graphs demonstrate the proportion of pro-inflammatory and anti-inflammatory lymphocytes in the vehicle groups and those treated with Etanercept at 12 mg/Kg for both strains. Statistical significance of intergroup comparisons: ns, non-significant; * *p* < 0.05. Mean ± SEM.

Flow cytometric analysis of dissociated cell suspensions obtained from the lumbar spinal cord (L4–L6) revealed a reduction in pro-inflammatory cell populations in Etanercept-treated MDX mice. Vehicle-treated MDX animals exhibited a 47.32% increase in GFAP^+^/TNF-α^+^ cells compared to C57BL/10 controls (*p* < 0.05). This difference was no longer significant following Etanercept treatment, with the interstrain difference reduced to 4.60% (Figure 2A).

In addition, a significant reduction (43.44%; *p* < 0.05) was observed in pro-inflammatory T helper (Th1) lymphocytes in Etanercept-treated MDX mice compared to vehicle-treated MDX animals. No statistically significant differences were detected among groups for anti-inflammatory Th2 lymphocytes (Figure 2B).

### 3.2. TNF-α Blockade Attenuates Astroglial and Microglial Reactivity in the Spinal Cord

**Figure 3:**
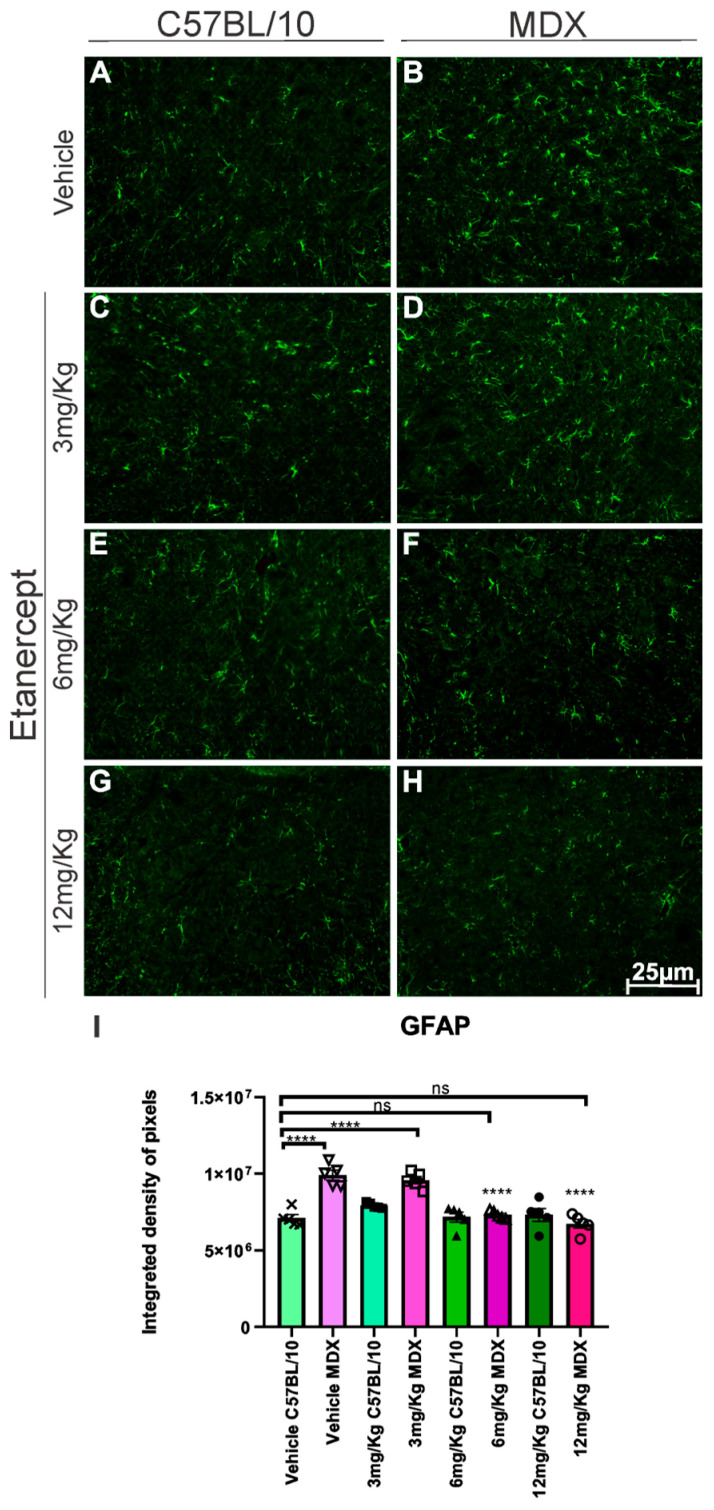
Anti-GFAP immunohistochemistry of cross-sections of the lumbar enlargement at 20× magnification. (**B**,**D**) Elevated expression in MDX animals compared to C57BL/10 (**A**,**C**,**E**,**G**). (**F**,**H**) Positive regulation of expression following treatment at doses of 6 and 12 mg/Kg. Scale bar: 25 μm. (**I**) Graph of GFAP immunohistochemistry in lumbar enlargement cross-sections, with quantification of the integrated density of pixels and statistical significance of intergroup comparisons: ns, non-significant; **** *p* < 0.0001. Mean ± SEM.

Immunohistochemical analysis of GFAP, a marker of astroglial reactivity, on the lumbar enlargement section from both strains revealed an approximately 40% increase in GFAP expression in MDX mice compared to C57BL/10 controls (*p* < 0.0001; Figure 3A,B,I). Treatment with Etanercept at 3 mg/Kg did not exhibit a statistically significant reduction in GFAP immunoreactivity compared to vehicle-treated MDX animals. In contrast, the 6 and 12 mg/Kg doses resulted in a significant decrease in GFAP expression (*p* < 0.0001), corresponding to reductions of approximately 25% and 33%, respectively (Figure 3B,F,H,I).

**Figure 4:**
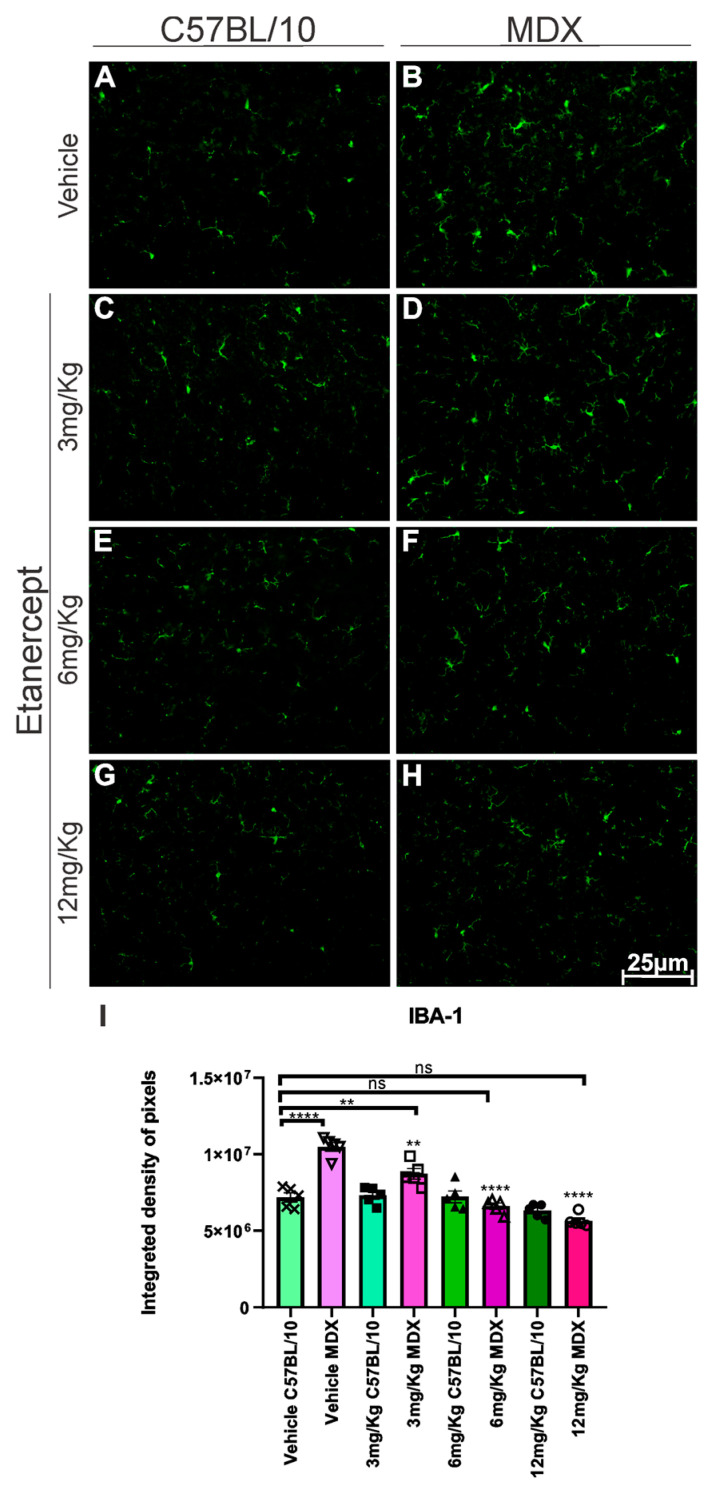
IBA-1 immunohistochemistry of lumbar enlargement cross-sections at 20× magnification. (**A**,**C**,**E**,**G**) Regulated expression in C57BL/10 animals. (**B**,**D**) Overexpression in MDX animals. (**F**,**H**) Positive regulation of expression in MDX animals after treatment with doses of 6 and 12 mg/Kg. Scale bar: 50 μm. (**I**) Graph quantifying IBA-1 expression in lumbar enlargement cross-sections. A difference is observed between the BL10 and MDX vehicle groups that decreases in animals treated with 3 mg/Kg and is not reproduced in animals treated with 6 and 12 mg/Kg. Quantification of the integrated density of pixels and statistical significance of intergroup comparisons: ns, non-significant; ** *p* < 0.01; **** *p* < 0.0001. Mean ± SEM.

Regarding microglial reactivity, IBA-1 immunolabeling was increased by approximately 48% in MDX mice compared to C57BL/10 animals (*p* < 0.0001; Figure 4A,B,I). Etanercept produced reductions in IBA-1 expression relative to their respective vehicle-treated MDX groups. The 3 mg/Kg dose resulted in a 17.71% reduction (*p* < 0.01; Figure 4B,D,I), while the 6 and 12 mg/Kg doses produced more pronounced decreases of approximately 37% and 46%, respectively (*p* < 0.0001; Figure 4B,F,H,I).

### 3.3. TNF-α Blockade Preserves Synaptic Inputs to Spinal Motoneurons in MDX

**Figure 5:**
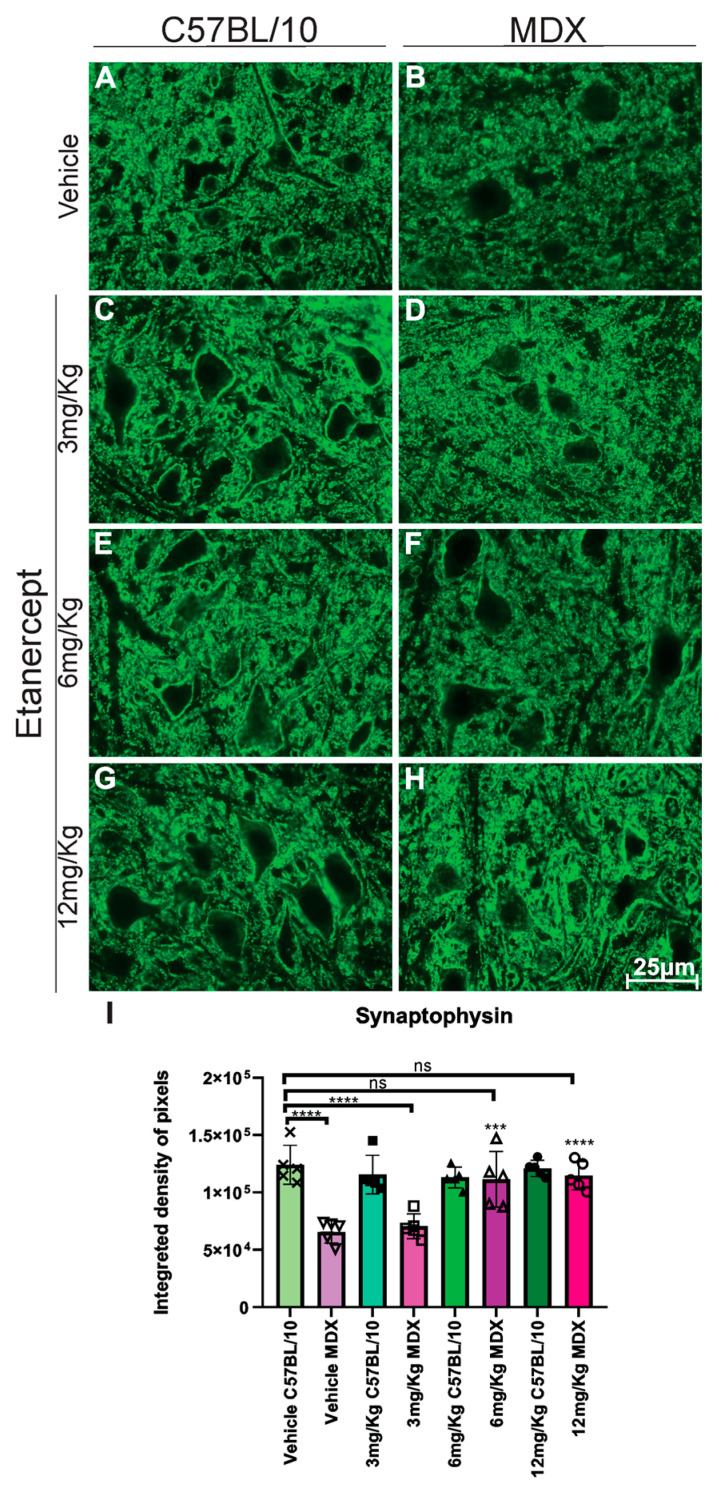
Immunohistochemistry for synaptophysin labeling in cross-sections of the lumbar enlargement at 40× magnification. (**A**,**C**,**E**,**G**) C57BL/10 animals with no significant alterations. (**B**,**D**) Reduced synaptophysin expression around motoneurons in MDX mice. (**F**,**H**) Preserved synaptic inputs following Etanercept administration in MDX mice. Scale bar: 25 μm. (**I**) Quantification of synaptophysin immunolabeling in the lumbar enlargement (L4–L6), based on the analysis of 8 points on the motoneurons surface, demonstrating the preservation of presynaptic input staining after Etanercept treatment in MDX mice. Quantification of the integrated density of pixels and statistical significance of intergroup comparisons: ns, non-significant; *** *p* < 0.001; **** *p* < 0.0001. Mean ± SEM.

Synaptophysin labeling on the motoneuron surface in MDX mice revealed a reduction in MDX mice, with untreated animals exhibiting an approximately 48% decrease compared to C57BL/10 controls (Figure 5). Etanercept treatment partially preserved synaptophysin expression in MDX mice in a dose-dependent manner. Animals treated with 6 mg/kg and 12 mg/Kg showed significant increases in synaptophysin labeling compared to vehicle-treated MDX mice (*p* < 0.001 and *p* < 0.0001, respectively), reaching approximately 56% and 83% of C57BL/10 control levels (Figure 5B,F,H,I).

**Figure 6:**
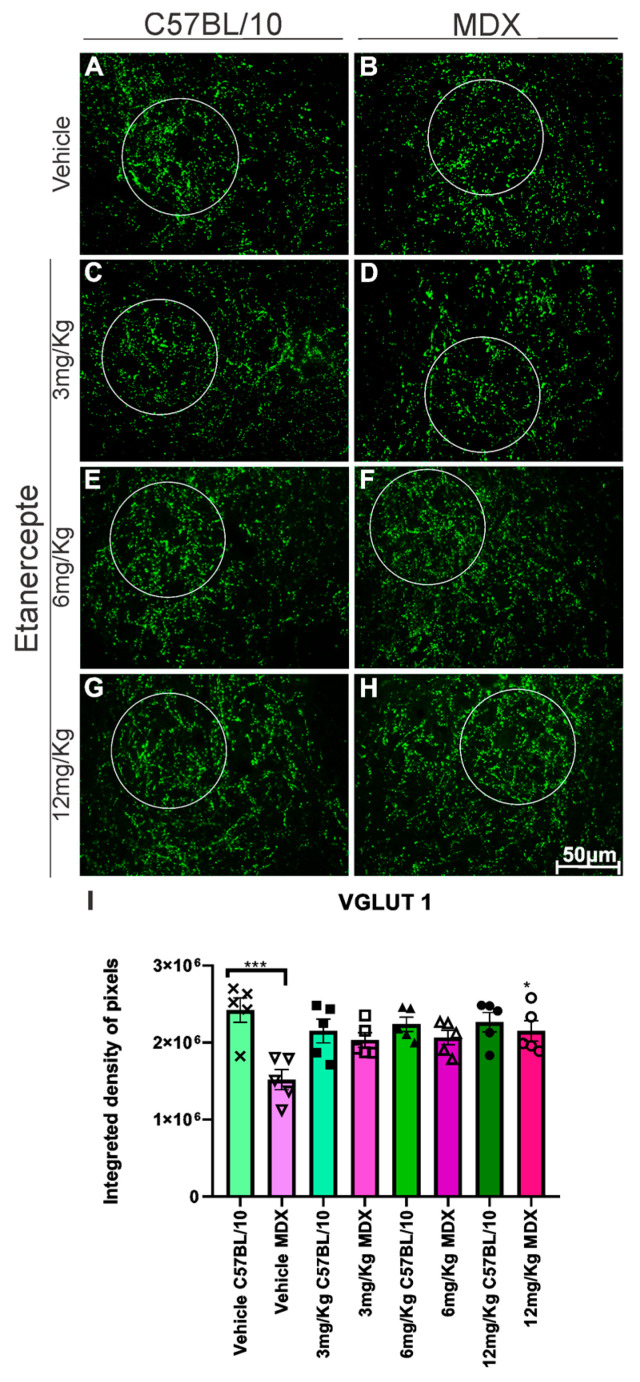
Anti-VGLUT1 immunohistochemistry in cross-sections of the lumbar enlargement at 20× magnification. White circles indicate representative regions of interest (ROIs) containing motoneuron-rich areas selected for quantitative analysis. (**A**,**C**,**E**,**G**) C57BL/10 animals showing no significant alterations. (**B**) Reduced VGLUT1 expression in MDX mice. (**D**,**F**) No significant changes following Etanercept administration at doses of 3 and 6 mg/kg in MDX mice. (**H**) Significant increase in VGLUT1 expression following treatment with 12 mg/kg compared with vehicle-treated MDX mice. Scale bar: 50 μm. (**I**) Quantification of anti-VGLUT1 immunolabeling in lumbar enlargement cross-sections comparing both strains across doses and MDX mice relative to their vehicle-treated group. Integrated density of pixels quantification and statistical significance of intergroup comparisons: ns, non-significant; * *p* < 0.05; *** *p* < 0.001. Data are presented as mean ± SEM.

Immunolabeling for vesicular glutamate transporter 1 (VGLUT1) revealed a significant reduction in expression in MDX mice compared to C57BL/10 controls. Etanercept treatment at 6 mg/kg showed a trend toward increased VGLUT1 expression in MDX mice (*p* = 0.0687); however, this did not reach statistical significance when compared with vehicle-treated MDX animals. In contrast, treatment with 12 mg/kg resulted in a significant increase in VGLUT1 expression (50.6%, *p* < 0.05) relative to vehicle-treated MDX mice (Figure 6B,F,H,I).

**Figure 7:**
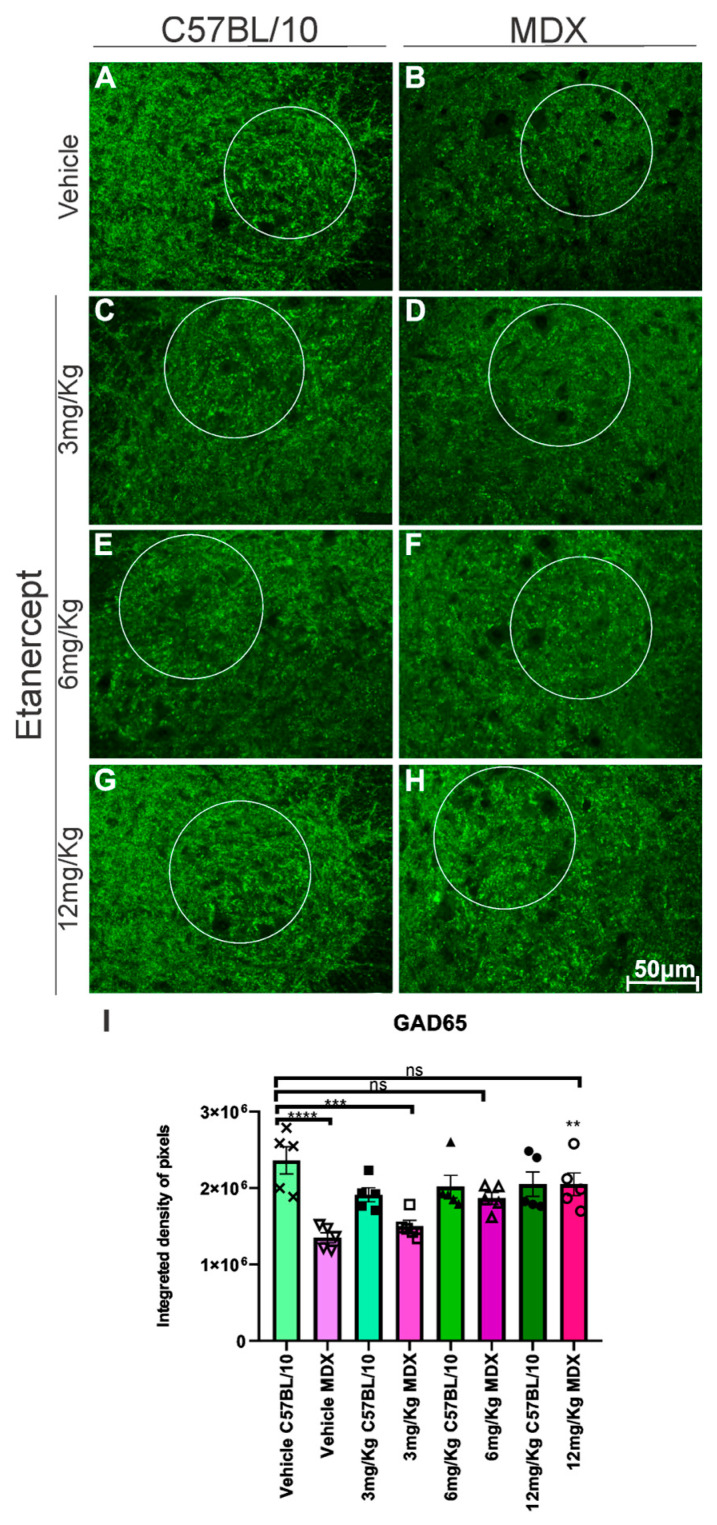
GAD-65 immunohistochemistry in cross-sections of the lumbar enlargement at 20× magnification in a defined area. White circles indicate representative regions of interest (ROIs) containing motoneuron-rich areas selected for quantitative analysis. (**A**,**C**,**E**,**G**) C57BL/10 animals showing no significant alterations. (**B**,**D**,**F**,**H**) Positive regulation in expression after treatment with Etanercept in MDX mice. Scale bar: 50 μm. (**I**) Graph of GAD-65 immunohistochemistry in lumbar enlargement cross-sections in a defined area, with quantification of the integrated density of pixels and statistical significance of intergroup comparisons: ns, non-significant; ** *p* < 0.01; *** *p* < 0.001; **** *p* < 0.0001. Mean ± SEM.

GAD65 expression was significantly reduced in MDX mice compared to C57BL/10 controls, with a 39% decrease observed in the ventral horn region containing spinal motoneurons (*p* < 0.0001). Etanercept-treated MDX mice exhibited an upregulation of GAD65 expression in a dose-dependent manner. While the increase at 3 mg/kg was modest and not statistically significant (11%), more pronounced increases were observed at 6 mg/kg (41%, non-significant) and 12 mg/kg, with the latter reaching statistical significance (42.8%, *p* < 0.01) compared to vehicle-treated MDX animals (Figure 7).

### 3.4. Qualitative Improvement of Skeletal Muscle Morphology Following TNF-α Blockade

**Figure 8:**
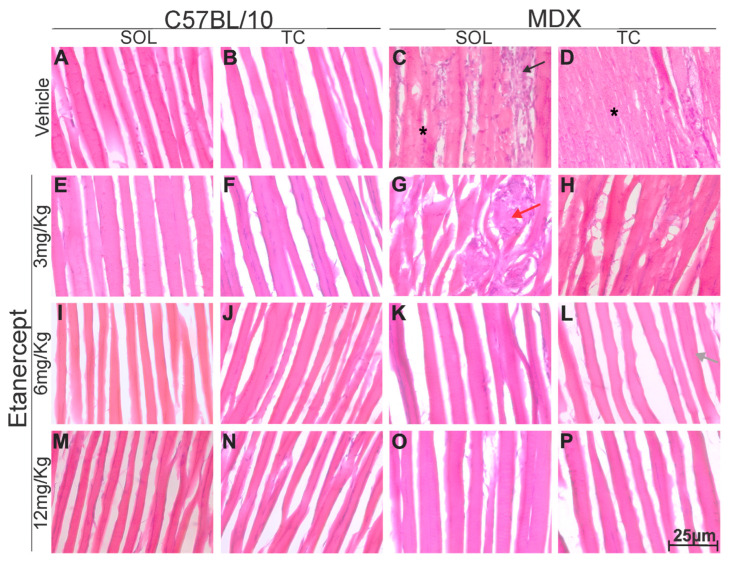
Longitudinal sections of soleus (SOL) and tibialis cranialis (TC) muscles stained with H&E from control (C57BL/10) and dystrophic (MDX) strains, 40× magnification. (**A**,**E**,**I**,**M**) Soleus muscle of C57BL/10 with healthy fibers, no inflammatory infiltrate, homogeneous staining, and peripheral nuclei. (**B**,**F**,**J**,**N**) Tibialis cranialis of C57BL/10 showing the same healthy pattern. (**C**) MDX soleus with deformed fibers (*), inflammatory infiltrates (black arrow), and marked degeneration. (**D**) MDX tibialis cranialis with similar degeneration and infiltration. (**G**) MDX soleus after 3 mg/Kg treatment showing persistent degeneration, fiber deformation, and infiltration (red arrow). (**H**) MDX tibialis cranialis after 3 mg/Kg with similar alterations. (**K**) MDX soleus after 6 mg/kg showing reduced infiltrates, more preserved fibers, and less degeneration. (**L**) MDX tibialis cranialis after 6 mg/Kg with reduced infiltrates, more preserved fibers, less degeneration, and some central nuclei (gray arrow). (**O**) MDX soleus after 12 mg/Kg showing further reduction in infiltrates, well-preserved fibers, and minimal degeneration. (**P**) MDX tibialis cranialis after 12 mg/Kg with similar improvements. Overall, higher Etanercept doses resulted in more preserved morphology and reduced pathological features compared to vehicle-treated MDX muscles.

According to expectation, morphological differences between the C57BL/10 and MDX strains were observed. Vehicle-treated MDX animals and MDX animals treated with 3 mg/Kg exhibited myodegeneration, characterized by a significant number of infiltrates, with muscle fibers surrounded by immune cells and displaying irregular morphology (Figure 8C,D,G,H). In several regions, deformed fibers and heterogeneous staining patterns were observed, including the presence of necrotic fibers containing basophilic inflammatory cells (Figure 8C,D,G,H).

In contrast, MDX mice treated with Etanercept at doses of 6 and 12 mg/kg showed a marked reduction in inflammatory infiltrates, along with the absence of large degenerative areas containing necrotic fibers and basophilic inflammatory cells. Treated animals also exhibited fewer fibers with centralized nuclei and a greater proportion of morphologically preserved fibers compared to vehicle-treated MDX mice (Figure 8 K,L,O,P).

### 3.5. Functional Improvement in MDX Mice Following TNF-α Blockade

After two weeks of evaluation using the CatWalk system, quantitative gait analysis was performed to test whether Etanercept treatment promoted functional recovery in MDX mice. Parameters were analyzed in a hierarchical manner, progressing from overall motor function to specific aspects of gait coordination and paw–ground interaction.

**Figure 9:**
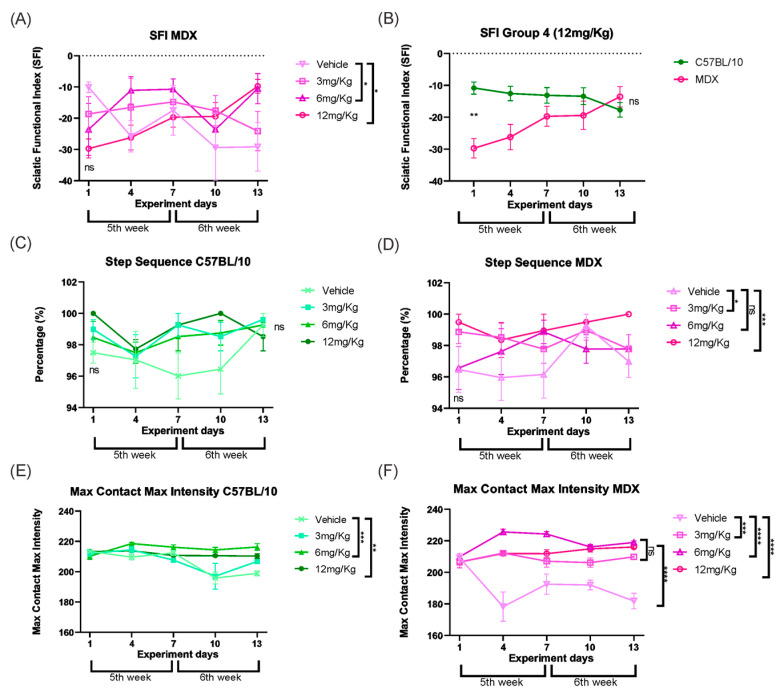
(**A**) SFI of the MDX strain in which the groups start without a significant difference be-tween them and, subsequently, the doses of Etanercept 6 and 12 mg/Kg show significant functional gain. (**B**) SFI of group 4 (12 mg/Kg) showing the development of the C57BL/10 and MDX animals throughout treatment with the statistical difference between them on the first day, without treatment, and on the last day of treatment. (**C**) Step sequence of the C57BL/10 strain shows that there was no significant difference between the groups at the beginning and end of the experiment, as expected. (**D**) Step sequence of the MDX strain showing a functional gain at doses of 3 and 12 mg/Kg. (**E**) Max Contact Max Intensity in C57BL/10 mice, showing maintenance of values in Etanercept-treated groups throughout the experimental period. (**F**) Max Contact Max Intensity in MDX mice, demonstrating stabilization in Etanercept-treated groups compared to the reduction observed in vehicle-treated animals. Statistical significance of intergroup comparisons: ns, non-significant; * *p* < 0.05; ** *p* < 0.01; *** *p* < 0.001, **** *p* < 0.0001. Mean ± SEM.

First, global motor performance was assessed using the sciatic nerve functional index. MDX mice treated with Etanercept at doses of 6 and 12 mg/kg showed a significant improvement compared to vehicle-treated MDX mice (*p* < 0.05), corresponding to functional gains of 63.3% and 74.1%, respectively (Figure 9A). Importantly, longitudinal analysis confirmed a treatment-dependent effect, as MDX mice receiving 12 mg/kg exhibited an 83.77% improvement between the first and final days of evaluation (*p* < 0.001; Figure 9B).

To determine whether these improvements were associated with changes in locomotor coordination, step sequence patterns were subsequently analyzed. As expected, C57BL/10 mice showed no differences between the initial and final evaluations (Figure 9C). In contrast, MDX mice displayed significant improvements in step sequence following Etanercept treatment at doses of 3 mg/kg (*p* < 0.05) and 12 mg/kg (*p* < 0.001) compared to vehicle-treated MDX mice (Figure 9D).

Finally, the interaction between the paw and the ground was evaluated by analyzing the maximum contact intensity. MDX mice treated with Etanercept showed constant values for this parameter at all doses, with similar effects among all doses at the end of the experiment, whereas vehicle-treated MDX mice exhibited a progressive decline throughout the experimental period (Figure 9E,F).

**Figure 10:**
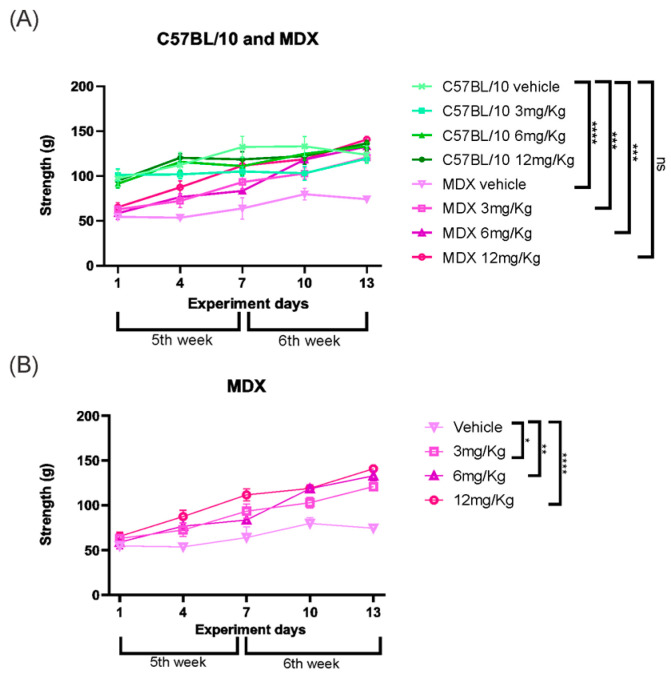
(**A**) Grip strength results showing strength gains in MDX-treated animals, particularly on the 6 and 12 mg/Kg doses. (**B**) Grip strength in grams of the MDX animals showing a gain in strength in all the groups treated with the dose of Etanercept. Statistical significance of intergroup comparisons: ns, non-significant; * *p* < 0.05; ** *p* < 0.01; *** *p* < 0.001; **** *p* < 0.0001. Mean ± SEM.

Grip strength analysis revealed a significant difference between strains in vehicle-treated groups, with MDX mice exhibiting a 33.12% reduction in strength compared to C57BL/10 controls (*p* < 0.0001). This difference was no longer observed when comparing vehicle-treated C57BL/10 mice with Etanercept-treated MDX animals. Vehicle-treated MDX mice displayed a consistently reduced strength pattern relative to treated groups, which showed significant improvements. Etanercept treatment increased muscle strength in MDX mice at all tested doses, with improvements of 37.17% at 3 mg/kg (*p* < 0.05), 37.22% at 6 mg/kg (*p* < 0.01), and the most pronounced effect observed at 12 mg/kg (63.89%, *p* < 0.0001) compared to vehicle-treated MDX animals (Figure 10).

## 4. Discussion

Duchenne muscular dystrophy (DMD) is a severe and incurable disease characterized by progressive skeletal muscle atrophy, and cardiac and respiratory dysfunction, generally culminating in death between the third and fourth decades of life [27]. It is now recognized that the severity of DMD goes beyond muscle impairment, also involving changes in the central and peripheral nervous systems [13,27]. In this context, systemic inflammation and neuroinflammation, particularly mediated by TNF-α, emerge as central axes in the progression of the disease.

Initially, flow cytometry analyses revealed changes in proinflammatory T helper (Th1) lymphocyte profiles and TNF-α expression, corroborating studies demonstrating the involvement of the adaptive immune system in the pathophysiology of DMD. In neurodegenerative diseases, CD4+ T cells can infiltrate the central nervous system and critically modulate microglial activation and subsequent neuronal damage [28]. In addition, both CD4+ and CD8+ lymphocytes have been associated with the progression of dystrophin-related dystrophies, with faster disease progression correlated with higher numbers of CD49, CD4+, and CD8+ cells [29,30]. The modulation of these inflammatory profiles after treatment with Etanercept reinforces the hypothesis that TNF-α blockade acts as a central mechanism in reducing systemic and neuroimmune inflammation.

As a direct consequence of this inflammatory modulation, we evaluated the effects of treatment on glial activation in the spinal cord. It is well established that lesions in the nervous system induce activation of astrocytes and microglia in response to degeneration [31,32]. TNF-α exerts a direct pro-inflammatory effect on astrocytes, promoting astrogliosis [33], and prolonged exposure compromises the survival of these cells [34]. The observed reduction in pro-inflammatory astrocytes (GFAP+; TNF-α+) and microglial activation (IBA-1+) after treatment with Etanercept can be attributed to its structure as a fusion protein formed by two copies of the human TNF-α receptor linked to the Fc portion of human IgG1, preventing the interaction of endogenous TNF-α with its receptors and, consequently, inhibiting its pro-inflammatory activity [35,36]. It is understood that a less inflammatory spinal cord environment is essential for the preservation of synaptic homeostasis.

In this scenario, the reduction in gliosis provides an underlying mechanism for findings related to synaptic connectivity. Synaptophysin is a glycoprotein present in the presynaptic terminals of excitatory and inhibitory neurons, widely used as a marker of neuronal connectivity [37]. In MDX mice, there is a significant reduction in alpha motoneuron innervation, associated with a decrease in corticospinal tract fibers [38,39], together with the loss of primary afferents and other synaptic contacts, resulting in less coverage of neuronal cell bodies in the spinal cord [13]. In the present study, the increase in synaptophysin expression after treatment with Etanercept (6 and 12 mg/kg) suggests that the attenuation of inflammation and glial activation contributes to the preservation and stability of spinal synapses.

In addition, we analyzed specific components of excitatory and inhibitory neurotransmission. Vesicular glutamate transporter 1 (VGLUT-1) is responsible for packaging glutamate into synaptic vesicles, and its availability is crucial for the effectiveness of synaptic transmission [40], with its overexpression associated with potentiation of neurotransmission [41,42]. In DMD, repeated cycles of muscle degeneration lead to the loss of muscle spindle innervation and degeneration of intrafusal innervation; consequently, primary afferents from the dorsal root ganglia are lost in the spinal cord, especially in Rexed’s lamina IX [13,43]. The improvement in VGLUT-1 immunostaining after treatment with Etanercept suggests preservation of these proprioceptive afferents, possibly mediated by the reduction of the inflammatory process.

With regard to inhibitory neurotransmission, glutamic acid decarboxylase (GAD-65) is essential for GABA synthesis and for maintaining the excitatory–inhibitory balance. Antibodies against GAD are associated with several neurological syndromes [44], and the regulation of their expression appears to be an adaptive mechanism in response to stressors [45,46]. Although little explored in muscular dystrophies, it is known that changes in dystrophin expression affect neuronal excitability, promoting an imbalance between excitatory and inhibitory synapses [47,48]. Thus, the low expression of GAD-65 observed in MDX mice treated with vehicle may reflect this imbalance, while its recovery after TNF-α blockade suggests partial restoration of synaptic homeostasis.

Alongside central changes, muscle degeneration is a key feature of DMD. Hematoxylin and eosin (H&E) staining allows for the evaluation of the overall architecture of muscle tissue, highlighting myofibers, inflammatory cells, connective tissue, and adipose tissue [49]. It is well established that muscular dystrophies cause significant structural damage to the muscles, with degenerating fibers surrounded by cellular infiltrates already visible in the first weeks of life in MDX mice [20,50]. In the present study, the tibialis cranialis and soleus muscles of six-week-old MDX mice showed extensive areas with cellular infiltrates and necrotic fibers, reflecting a chronic inflammatory process.

This muscle inflammation results from the continuous release of cytoplasmic content from dystrophic fibers, activating the innate immune system and perpetuating the recruitment of inflammatory cells [51]. Repeated cycles of degeneration and inefficient repair prolong the inflammatory process, culminating in fibrosis, connective and adipose tissue deposition, and progressive muscle loss [50]. In addition, sarcolemmal ruptures lead to Ca^2+^ homeostasis dysfunction, mitochondrial overload, increased production of reactive oxygen species, and mitochondria-dependent cell necrosis [52]. In this context, interventions capable of modulating inflammation and improving mitochondrial function may attenuate muscle degeneration [53].

Treatment with Etanercept promoted evident improvement in muscle morphology in MDX mice, especially at doses of 6 and 12 mg/kg, suggesting that TNF-α blockade reduces inflammation and muscle degradation, contributing to the preservation of dystrophic muscle. These findings reinforce the potential of highly specific anti-inflammatory drugs as a relevant therapeutic alternative, possibly more advantageous than the non-specific corticosteroids currently used, such as prednisolone and deflazacort [20].

These structural and molecular changes are directly reflected in functional outcomes. Previous studies have demonstrated motor deficits in MDX animals even in the absence of obvious neural lesions [13], as well as impairments associated with inflammation and immune cell infiltration [54]. In the present study, animals treated with Etanercept showed a significant improvement in motor performance in some parameters analyzed and stabilization in others, unlike what was observed in untreated vehicle animals, as assessed by the CatWalk system, as well as an increase in grip strength, especially at higher doses. These results indicate that functional recovery does not depend on a single axis; instead, it results from the convergence of reduced inflammation, preservation of spinal synaptic connectivity, and improved muscle integrity.

Finally, although Etanercept has limitations in crossing the blood–brain and blood–bone marrow barriers due to its high molecular weight [55], it is plausible that peripheral blockade of TNF-α, particularly in muscle and peripheral nerve endings, triggers retrograde effects capable of modulating spinal motoneuron activity and the spinal cord microenvironment. Furthermore, it cannot be ruled out that low levels of central penetration or indirect action on the spinal vasculature may be sufficient to contribute to the observed effects. Therefore, the results point to an integrated mechanism involving muscle, peripheral nerve, and spinal cord, reinforcing the systemic interpretation of DMD and the therapeutic relevance of TNF-α blockade.

## 5. Conclusions

The present results indicate that treatment with Etanercept, particularly at doses of 6 and 12 mg/kg, attenuates inflammatory responses in MDX mice and is associated with preservation of synaptic organization and reduced retrograde degenerative changes in the central nervous system. These effects were accompanied by clear improvements in muscle morphology, along with reduced inflammatory infiltrates and better preservation of muscle fibers. Importantly, such structural changes were reflected in functional outcomes, as treated animals showed improved motor performance in gait analysis and increased grip strength. Together, these findings suggest that TNF-α blockade contributes to functional preservation in MDX mice through combined effects on inflammation, neuromuscular integrity, and spinal synaptic stability.

## Figures and Tables

**Table 1:** Experimental groups and techniques used.

Strain	Treatment/Drug Dosage	Evaluation				
		Functional		Morphological		
		Catwalk	Grip Strength	Immunohistochemistry	H&E	Flow Cytometry
MDX *	Vehicle/0.08% methyl cellulose	10	10	5	10	5
C57BL/10 *	Vehicle/0.08% methyl cellulose	10	10	5	10	5
MDX	3 mg/Kg Etanercept	5	5	5	5	-
C57BL/10	3 mg/Kg Etanercept	5	5	5	5	-
MDX	6 mg/Kg Etanercept	5	5	5	5	-
C57BL/10	6 mg/Kg Etanercept	5	5	5	5	-
MDX *	12 mg/Kg Etanercept	10	10	5	10	5
C57BL/10 *	12 mg/Kg Etanercept	10	10	5	10	5

* The number of mice required increased by a factor of two because of the need for specific procedures for different techniques.

## Data Availability

The data presented in this study are available upon request from the corresponding author. The data are not publicly available due to ethical and institutional restrictions.

## Abbreviations

The following abbreviations are used in this manuscript:

CEUA	Ethics Committee for the Use of Animals
CNS	Central nervous system
CONCEA	Brazilian Committee for Ethics in the Use of Animals
DAPC	Dystrophin-associated protein complex
DMD	Duchenne muscular dystrophy
GAD65	Glutamic acid decarboxylase
GFAP	Glial Fibrillary Acidic Protein
H&E	Hematoxylin and eosin
IBA-1	Ionized Calcium-Binding Adapter Molecule 1
JRA	Juvenile rheumatoid arthritis
PB	Phosphate buffer
PBS	Sterile phosphate-buffered saline
PMA	Phorbol 12-myristate 13-acetate
PNS	Peripheral nervous system
PRISMA	Referred Reporting Items of Systematic Reviews and Analysis
RA	Rheumatoid arthritis
TNF-α	Tumor necrosis factor alpha
VGLUT 1	Vesicular glutamate transporter 1

## Appendix A

### Appendix A.1

**Table A1:** Primary antibodies used in the research.

Antibody	Manufacturer	Host	Concentration
GFAP	Santa Cruz	rabbit anti-human	1:750
IBA-1	Wako	rabbit anti-mouse	1:750
VGLUT 1	Synaptic Systems	rabbit anti-mouse	1:1000
GAD-65	Abcam	rabbit anti-mouse	1:750
Synaptophysin	Novus Biologicals	rabbit anti-mouse	1:1000

### Appendix A.2

**Table A2:** Antibodies used for the flow cytometry technique.

Cell Type	Alexa 488/FITC	PE	PE-Cy5	PE-Cy7	APC	APC-C
Lymphocytes	CD4			INF-γ	IL-4	CD3
Astrocytes	GFAP	IL-10	TNF-α	INF-γ	IL-4	
Microglia	DC45	IL-10	TNF-α	CD206	CD68	CD11b
